# Beyond Contact Angle: Reframing the Evaluation of Super-Liquid-Repellent Materials for Real Food Environments

**DOI:** 10.3390/nano16181140

**Published:** 2026-09-10

**Authors:** Jia Xia, Jian Li, Weifeng Jin

**Affiliations:** 1 School of Materials Science and Engineering, Jiangsu University, Zhenjiang 212013, China; xiajia041127@163.com; 2 School of Mechanical Engineering, Jiangsu University, Zhenjiang 212013, China; jinweifeng2009@yeah.net

**Keywords:** super-liquid-repellent materials, food-contact materials, dynamic evaluation, interfacial fouling

## Abstract

Super-liquid-repellent materials hold significant promise for minimizing food residue, suppressing interfacial fouling, and enhancing the cleanability of food-contact surfaces. However, prevailing evaluation paradigms—centered on static contact angle, roll-off angle, and dry abrasion—fail to forecast long-term service performance in complex food-processing environments. Unlike idealized probes, real food matrices comprise proteins, polysaccharides, lipids, surfactants, and microbiota, driving interfacial behavior that is time-dependent, multicomponent-coupled, and dynamically evolving. Consequently, traditional static metrics are inadequate across temporal, chemical, and mechanical dimensions. Furthermore, while “fluorine-free,” “edible,” or “bio-based” labels offer design cues, they cannot substitute for rigorous, lifecycle-resolved assessments encompassing fabrication, aging, migration, and end-of-life impacts. This review delineates the fundamental mismatch between current evaluation frameworks and operational food environments, exposing latent safety and sustainability risks obscured by superficial green claims. We subsequently discuss a dynamic evaluation framework featuring multidimensional metrics and a tiered screening workflow, shifting the paradigm from endpoint-focused assessment to a process-based evidentiary chain. Finally, we outline future trajectories, emphasizing the transition from passive repellency to active fouling modulation and the co-design of performance, safety, and sustainability. This work provides a conceptual blueprint for updating evaluation standards and accelerating the industrial translation of food-contact super-liquid-repellent materials.

## 1. Introduction

In the food industry, the adherence of high-viscosity or complex-composition foods to packaging, conveyance systems, and processing equipment results in significant product loss and poses substantial risks of cross-contamination and microbial growth [1,2,3,4]. Drawing inspiration from the lotus leaf effect, super-liquid-repellent surfaces have emerged as a promising strategy for enhancing food safety and extending shelf life [5,6,7,8]. However, the reliable characterization of these surfaces hinges on the selection of appropriate performance metrics, rendering this choice critically important.

Super-liquid-repellent surfaces are typically defined as interfaces exhibiting an apparent contact angle > 150° and a roll-off angle < 10° for water (superhydrophobic) or relevant food liquids (superamphiphobic) [9,10,11]. These surfaces are architected through two complementary strategies: (i) the creation of hierarchical micro–nano roughness (e.g., re-entrant structures, nanopillar arrays, nanoparticle assemblies, etched textures) that traps air to sustain the Cassie–Baxter state; and (ii) chemical modification with low-surface-energy components to minimize interfacial adhesion. Representative architectures include T-shaped overhangs, porous coatings, slippery liquid-infused porous surfaces (LIPS/SLIPS), and polymer-brush-grafted interfaces. Currently, the performance evaluation of super-liquid-repellent materials predominantly relies on static contact angle, roll-off angle, and sandpaper abrasion tests [12,13,14,15]. While these metrics facilitate initial material screening, they primarily capture instantaneous wetting behavior under idealized laboratory conditions [16,17,18,19,20,21]; consequently, they offer limited predictive value regarding long-term durability in real food-processing environments [22,23,24]. Such environments involve complex matrices containing proteins, polysaccharides, lipids, salts, surfactants, and microorganisms [25,26,27,28,29,30], which collectively challenge surface integrity. Beyond these methodological limitations, the environmental claims frequently advanced in the literature warrant critical scrutiny. Although labels such as “fluorine-free” [31,32,33], “edible” [34,35,36], and “bio-based” [37,38,39] are routinely invoked to signify ecological safety and sustainability, they do not inherently guarantee low environmental impact or negligible biological risk [40,41,42,43,44].

**Figure 1:**
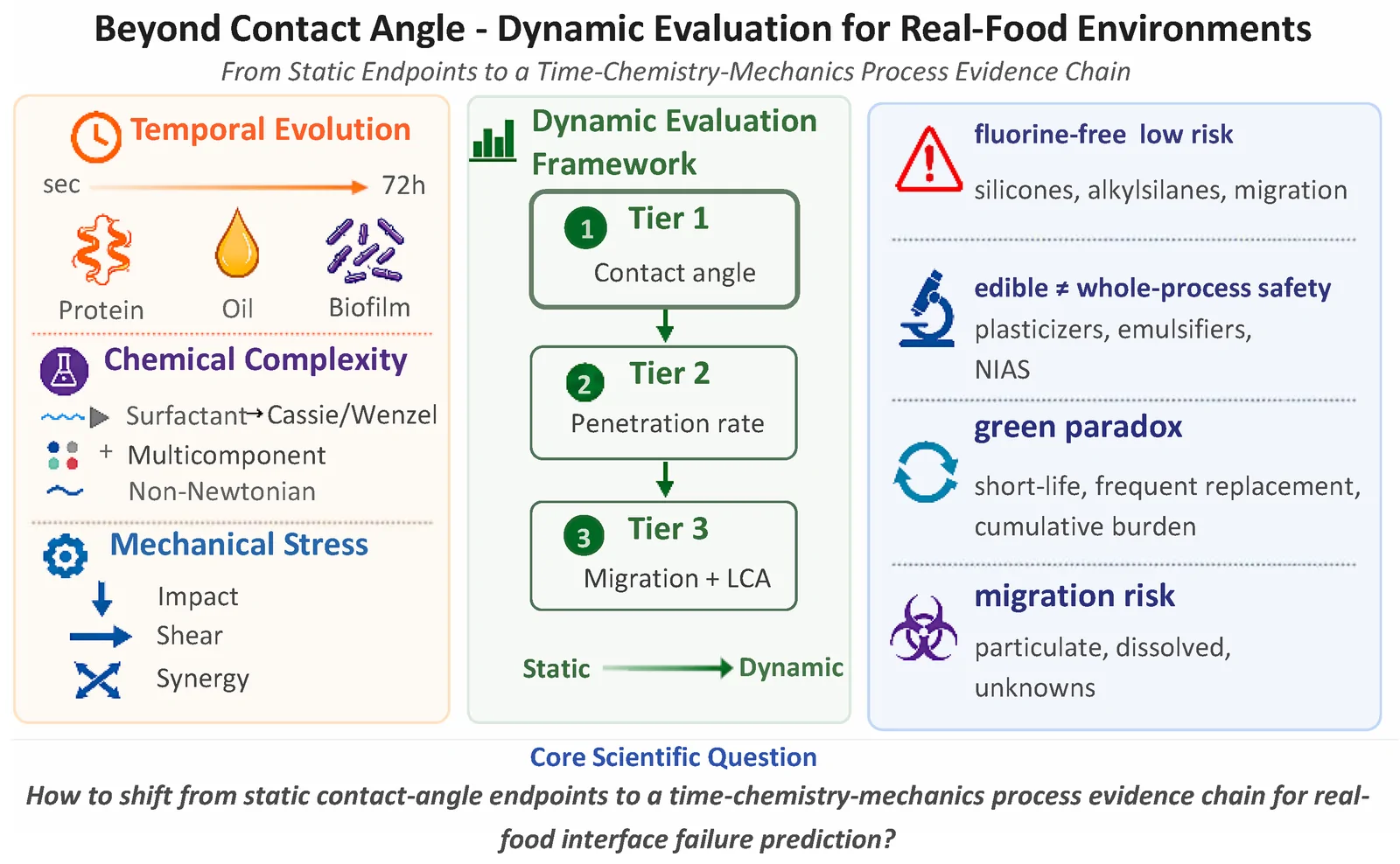
Dynamic evaluation framework for super-liquid-repellent materials in real food environments. The framework integrates temporal fouling evolution, chemical interference, mechanical stress, hidden safety/sustainability costs, and tiered screening to shift evaluation from static contact-angle endpoints to a time–chemical–mechanical process-based evidence chain.

Motivated by these limitations, this review posits that research on super-liquid-repellent materials for food applications must move beyond merely verifying initial super-repellency. Instead, it should address the timing, mechanisms, and ramifications of material failure within real food environments—encompassing safety hazards and environmental consequences. Accordingly, this review first delineates the discrepancies between prevailing evaluation protocols and the complex realities of food processing across temporal, chemical, and mechanical dimensions. It then critically examines the often-overlooked fabrication burdens and migration risks associated with purportedly “green” materials. Building on this analysis, we propose a dynamic evaluation framework tailored to real food matrices, integrating multidimensional performance metrics with a tiered screening workflow (Figure 1). Finally, future perspectives are outlined, highlighting a paradigm shift from passive liquid repellency to the active management of interfacial fouling evolution.

## 2. Rethinking Performance Metrics: From Idealized Static Wetting to Fouling Dynamics in Food Matrices

### 2.1. Temporal Dimension: From Transient Repellency to Persistent Biofouling

**Figure 2:**
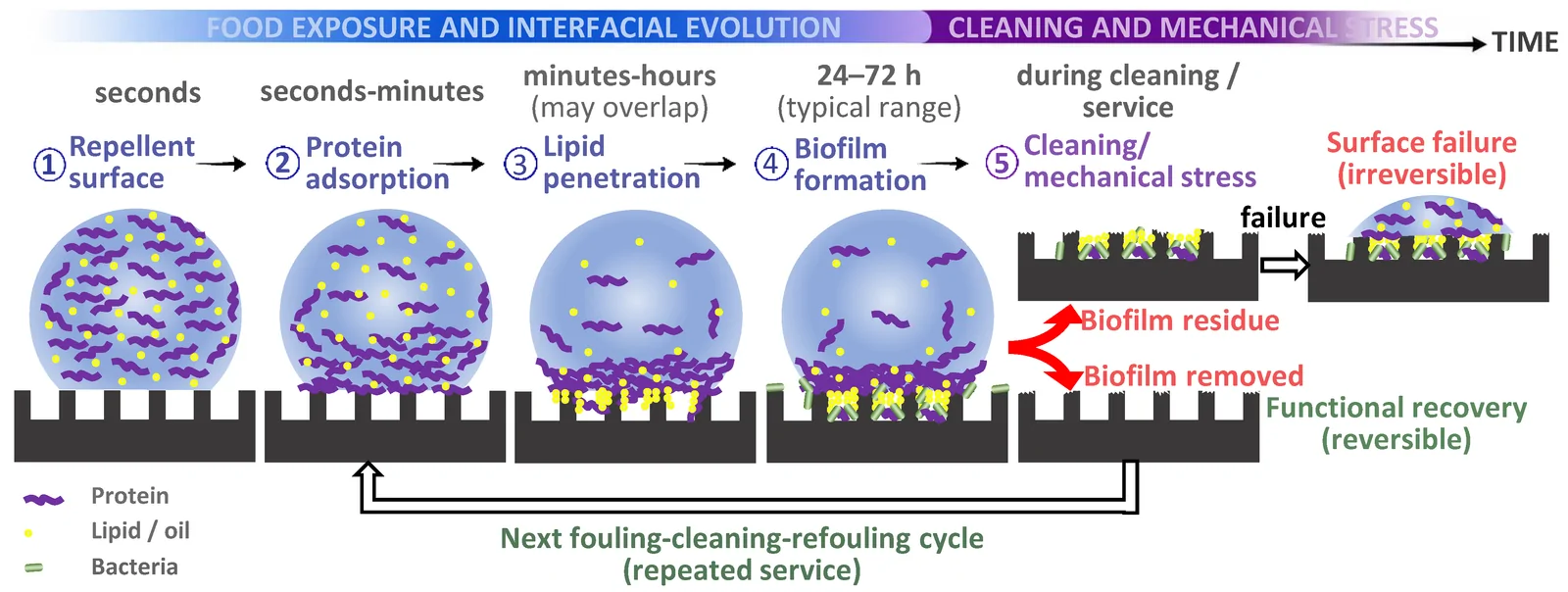
Time-dependent evolution from the pristine state through fouling and processing stresses toward failure or recovery.

Introducing super-repellent surfaces into the food industry requires attention not only to initial repellency but also to persistent biofouling. These surfaces typically feature abundant micro–nano architectures that promote a stable Cassie state at the liquid–solid interface [45], thereby yielding high apparent contact angles and low adhesion or roll-off resistance [46,47,48,49]. However, such metrics are predominantly established for simple model liquids (e.g., water and oils) and thus provide limited insight into the complex interfacial dynamics of food systems. In real food environments, fouling is not a binary “stick–nonstick” event but a time-dependent, multistage process governed by contact duration [50,51], interfacial composition [52,53], and external stresses [54,55]. Failure initiates not with an abrupt loss of contact angle but through sequential events: protein adsorption and rearrangement [56,57,58,59,60], capillary penetration of oils or lipids into micro–nano textures [61,62,63,64,65], displacement of the entrapped air cushion by surface-active components [66,67,68,69], and progressive microbial colonization culminating in biofilm maturation [70,71,72,73,74,75,76], as shown in Figure 2.

The temporal progression of each fouling type underscores the inadequacy of single-point measurements. Proteins adsorb within seconds to minutes, followed by slow reorientation and conformational rearrangement that transitions adhesion from reversible to irreversible over tens of minutes to hours [77,78,79]. Lipids and oils may simultaneously infiltrate surface textures via capillary action and subsequently crystallize upon cooling—a particularly critical pathway within the 4–25 °C range commonly encountered in food processing and storage [80,81,82,83,84,85]. Microbial fouling further exemplifies this temporal complexity: initial attachment progresses through microcolony formation to mature biofilm establishment [86,87,88], with key transitions typically occurring within 24–72 h [73]. Barreto et al. [58] employed in situ ATR-FTIR/2D-COS to monitor bovine serum albumin (BSA) adsorption on hematite surfaces, revealing a marked decrease in α-helix content concomitant with an increase in β-sheet structures. Consequently, surfaces exhibiting excellent initial repellency can fail rapidly under practical conditions due to protein anchoring, lipid entrapment, or biofilm overgrowth.

Beyond temporal evolution, the extent and irreversibility of protein–surface interactions are governed by four interrelated surface physicochemical properties: (1) Surface chemistry and functional group identity—hydrophilic or charged groups (–OH, –COOH, –NH_2_) promote strong binding, whereas low-surface-energy termini (–CH_3_, –CF_3_) minimize it; adsorption is a conformational competition where side-chain rearrangement can expose buried hydrophobic residues and amplify fouling [89,90]. (2) Surface charge and electrostatic matching—proteins (e.g., BSA, β-lactoglobulin) carry pH-dependent net charges; opposite charges accelerate deposition, while charge screening by food-matrix salts reduces adhesion [91,92]. (3) Hydration layer and water structure—a tightly bound interfacial water network (e.g., zwitterionic brushes, PEG, LIPS) imposes a desolvation penalty that prevents protein approach, whereas hydrophobic surfaces allow rapid penetration [93,94,95]. (4) Roughness and topography—hierarchical roughness increases contact area and creates topographic traps that immobilize proteins, yet in the Cassie state, entrapped air cushions reduce effective solid–liquid contact [96,97,98]. As proteins and surfactants displace the plastron over time, the same texture becomes a fouling reservoir inaccessible to cleaning agents.

These mechanistic complexities carry direct implications for evaluation methodology. Contact-angle retention after protein exposure is a convenient first-line indicator, but it is an indirect metric: a stable angle does not preclude the formation of a thin, tenacious protein monolayer that has already compromised functionality. Therefore, contact-angle retention should be complemented by direct adsorption quantification—e.g., QCM-D for real-time mass/viscoelastic changes, fluorescence labeling, XPS for elemental composition, or in situ spectroscopy (ATR-FTIR/2D-COS) for conformational analysis. A robust dynamic evaluation framework must integrate these orthogonal probes to resolve not only whether fouling occurs, but how and how irreversibly—thereby enabling targeted material design rather than empirical performance reporting. True antifouling efficacy must be defined by the duration for which irreversible fouling can be deferred, rather than the transient absence of adhesion.

### 2.2. Chemical Dimension: From Passive Repellency to Competitive Interfacial Adsorption

Conventional evaluation frameworks also suffer from excessive simplification regarding chemical complexity and operational dynamics. Most studies rely on pure water or single-oil phases [99,100,101] as probing liquids and loosely attribute surface failure to “reduced wettability”. In stark contrast, real food interfaces comprise multicomponent matrices—including proteins [102,103], polysaccharides [104,105], lipids [106,107], salt ions [108,109], and various surfactants [110,111]—where interfacial behavior is governed by competitive adsorption, dynamic interfacial tension fluctuations, and structural reorganization under the synergistic effects of flow-induced shear, thermal cycling, and cleaning agents [1,5,112]. For instance, surface-active amphiphiles can preferentially occupy interfacial sites, displacing the entrapped air cushion essential for the Cassie state [113,114,115]. Concurrently, the complex stress fields generated by viscoelastic food slurries during processing can alter hydrodynamic transport, enhancing the deposition flux of foulants toward the interface [112]. Fundamentally, interfacial performance in food systems is dictated by the kinetics of competitive adsorption and the viscoelastic response of the interfacial layer, rather than merely the initial apparent contact angle of an idealized droplet. Consequently, frameworks centered exclusively on static water contact angle measurements effectively reduce a multivariate interfacial phenomenon to a simplistic surface-energy problem, overlooking the mechanistic drivers of failure in real-world settings [1].

A critical failure mechanism frequently overlooked by static wetting assays is the disruption of the Cassie state by surface-active species. Upon diffusing to the solid–liquid interface, amphiphilic molecules preferentially adsorb, thereby reducing local interfacial free energy and compromising the thermodynamic stability of the air cushions entrapped within surface micro–nano architectures [113]. This process promotes an irreversible transition of droplets from the low-adhesion Cassie–Baxter state to the high-adhesion Wenzel state [113,116]. Shardt et al. [69] systematically examined the effect of surfactants on hydrophobic microstructured surfaces and demonstrated that, at sufficiently high concentrations, all test droplets collapsed into the Wenzel state, completely losing their gas-trapping capability. These findings indicate that emulsifiers, lipid-associated amphiphiles, and endogenous surfactants ubiquitous in real foods do far more than marginally lower the contact angle; they fundamentally alter the wetting regime. Consequently, reliance on pure-water contact angle measurements alone significantly overestimates the practical antifouling performance of super-repellent surfaces when exposed to complex, amphiphile-rich media such as dairy products, sauces, and high-fat formulations [69]. In light of this surfactant-mediated wetting transition, the evaluation focus for food-contact surfaces must advance beyond static single-droplet goniometry toward dynamic assessments of interfacial stability under realistic multicomponent and multirheological conditions.

### 2.3. Mechanical Dimension: Beyond Solid-on-Solid Abrasion to Fluid-Driven Erosion

Recently, sandpaper abrasion tests have been adopted to benchmark the mechanical durability of super-repellent surfaces; however, these protocols remain far from standardized [117,118,119]. Typical implementations involve 600–1000 grit sandpaper, applied loads of 50–100 g (corresponding to contact pressures of approximately 5–25 kPa), single-pass sliding distances of 10–20 cm, and cycle counts ranging from tens to several hundreds [120,121,122,123,124]. While such assays offer a convenient means of screening dry mechanical robustness, they are a poor proxy for the complex wear mechanisms encountered in food-processing environments. In real service, food-contact surfaces are subjected to a synergistic combination of flow-induced scouring, droplet impingement, thermal cycling, chemical attack from cleaning agents, and intermittent mechanical friction—not merely dry abrasion [125,126,127,128]. Critically, functional failure in these contexts is rarely governed by abrasive mass loss; instead, it is driven by collapse of the entrapped air plastron, capillary infiltration of process fluids into surface micro–nano architectures, and degradation of the chemically active outer layer [129,130,131]. Consequently, a surface that survives thousands of dry abrasion cycles may still fail catastrophically under modest operational stresses typical of dairy, beverage, or sauce production lines.

Mechanistically, the durability of super-liquid-repellent surfaces hinges on the preservation of an entrapped air plastron within surface micro–nano architectures. When external stimuli—such as hydrostatic pressure [132,133,134], droplet impact [135,136,137], or liquid shear [138,139,140]—exceed the interfacial capillary resistance, working liquid can impale the surface texture, triggering an irreversible transition from the Cassie–Baxter state to the high-adhesion Wenzel state. Forsberg et al. [61] directly visualized this Cassie-to-Wenzel transition under controlled hydrostatic-pressure conditions and demonstrated that both surface topography and applied pressure critically govern plastron destabilization. These results underscore that interfacial robustness is dictated not merely by a high initial contact angle, but by the surface’s capacity to sustain a non-wetted (air-retaining) configuration under realistic mechanical stresses. Consequently, reporting that a surface retains a contact angle > 150° after hundreds of dry abrasion cycles is insufficient to forecast its service life in food-processing lines. Without explicit assessment of liquid impalement under pressure and impact, such metrics offer little predictive value for performance during conveying, filling, or high-pressure cleaning operations.

In food-processing environments, mechanical degradation and fouling accumulation are not isolated events but act synergistically to undermine surface performance. Crucially, the ability to shed pure water droplets after short-term mechanical testing offers little insight into the progressive nanoscale accumulation of proteins, lipids, and microorganisms. Liu et al. [1] emphasized that adhesion and mechanical interlocking of complex food fluids are governed jointly by rheological behavior and surface topography—factors that current standardized tests fail to capture in a unified manner. Consequently, durability in real-world settings should be redefined: it is the capacity to sustain low adhesion and low residue across repeated cycles of fluid shear, fouling deposition, and cleaning, rather than merely retaining a high contact angle after dry abrasion. In essence, the mechanical evaluation of food-contact surfaces must evolve from a binary assessment of abrasion resistance to a holistic interrogation of interfacial functionality under realistic, multifactorial operating conditions [1,61].

Ultimately, the persistent mismatch between conventional static assays and real-world food environments stems from a fundamental misalignment in the object of evaluation. Static protocols merely quantify initial liquid repellency under idealized, contaminant-free conditions [20]. In contrast, application-relevant assessment must address when failure occurs, why it occurs, and whether functionality can be restored after cleaning. To bridge this gap, evaluation frameworks must evolve along three interdependent axes: temporally, by resolving the evolution from reversible adsorption to irreversible fouling; chemically, by capturing multicomponent competitive adsorption and interfacial restructuring; and mechanically, by quantifying interfacial stability under coupled pressure, impact, cleaning, and fouling accumulation. Only by integrating these multidimensional insights can the assessment of super-liquid-repellent surfaces transcend empirical wetting metrics and converge with the operational realities of the food industry.

## 3. Hidden Costs Behind Green Labels

### 3.1. Complexity of “Fluorine-Free” and “Edible” Labels

Equating “fluorine-free” status [141,142,143,144] with inherently low risk is scientifically untenable. The actual hazard profile of food-contact super-liquid-repellent surfaces is governed not merely by the absence of fluorinated compounds, but by the collective behavior of low-surface-energy components [6,118,123,145], polymeric binders [146,147,148], additives [149,150], and their post-service migration potentials. Notably, even within ostensibly fluorine-free frameworks, current strategies frequently rely on silica [151,152,153,154], long-chain alkylsilanes [155,156,157,158,159], or cross-linked resins [160,161,162] to preserve the requisite surface energy and micro–nano structural integrity. Consequently, “fluorine-free substitution” often represents a structural permutation rather than a comprehensive mitigation of persistence, mobility, or toxicological risk. Compounding this issue is the prevalence of non-intentionally added substances (NIAS) [163,164,165]—including synthetic by-products, thermal degradants, and trace impurities—which are inherent to complex coating formulations. These migrants can transfer into food matrices under processing conditions, yet current risk-assessment paradigms remain constrained by limited analytical coverage and quantification of unknown NIAS [42,43,166,167,168]. Therefore, evaluations that terminate at fluorine screening create a misleading narrative: substituting one class of chemicals of concern does not equate to a proportional reduction in systemic risk, nor does it guarantee functional safety under real-world food-processing stresses.

The “edible” designation must likewise be contextualized within the entirety of the fabrication chain [169,170,171]. While it legitimately signifies that certain base substrates are food-grade [172,173,174,175], it should not be misconstrued as a blanket certification of safety for the entire coating system or its manufacturing process [176,177,178]. Comprehensive reviews of edible films and coatings reveal that these matrices—typically comprising proteins, polysaccharides, and lipids—routinely necessitate the incorporation of plasticizers, emulsifiers, bioactive agents, and even nanofillers to tailor film formation, barrier properties, and mechanical integrity [41]. Consequently, safety assessments cannot be confined to the principal substrate; they must rigorously evaluate whether all auxiliary agents and composite constituents comply with food-contact regulatory standards.

Representative studies on super-liquid-repellent surfaces further underscore this nuance. For instance, liquid-repellent coatings derived from food-grade waxes (e.g., paraffin or beeswax) [179,180] often entail fabrication protocols involving ethanol-based dispersion, spray deposition, and thermal annealing. Similarly, so-called “food-grade” anti-adhesive formulations may integrate food-grade paraffin wax with PDMS-modified attapulgite clays [118,123,145,181] and food-grade silicone binders [143]. These cases demonstrate that a “food-grade” label applied to raw materials does not automatically confer safety upon the final processed article or the transformation pathways involved. The pertinent inquiries must instead target the migration profile of the composite system [182,183,184,185]: which specific components migrate under conditions of intended use, to what extent, and in what chemical speciation? Therefore, “fluorine-free” and “edible” are more appropriately framed as foundational design criteria or research starting points—rather than as definitive evidence of safety or sustainability [186,187,188].

### 3.2. Sustainability Costs During Fabrication

Sustainability is not solely dictated by the origin of raw materials; it is equally contingent upon the alignment between fabrication overhead and functional service life [189,190,191]. The environmental footprint of super-liquid-repellent surfaces is predominantly governed by the combined metrics of material consumption per fabrication cycle and the frequency of recoating interventions. Consequently, a coating formulated with natural feedstocks but reliant on solvent-assisted processing [192], energy-intensive thermal curing [193], or multistep deposition [194] may incur an annualized energy demand and waste burden comparable to—or even exceeding—those of a conventional, longer-lived alternative. Recent critical reviews in food packaging increasingly underscore this distinction: the descriptor “eco-friendly” more accurately reflects a nascent design objective than a validated outcome of comprehensive life cycle assessment (LCA) [40,195,196,197].

Empirical comparisons within the extant literature vividly illustrate this contention. Consider super-liquid-repellent coatings derived from food-grade waxes: while ostensibly benign in raw material sourcing and facile in laboratory preparation, their fabrication protocols routinely encompass energy-intensive steps such as ethanol-based spray deposition, thermal dissolution, cooling-induced precipitation, and thermal annealing [198,199,200,201]. To bolster thermal and mechanical robustness, researchers frequently implement structural augmentation strategies, including bilayer architectures or the incorporation of mineral fillers [202,203,204]. For instance, the natural/edible bilayer coating engineered by Wang and Zhao [205] retained an apparent contact angle > 150° following thermal exposure at 80 °C for 30 min or cryogenic storage at −20 °C for 24 h, demonstrating that deliberate structural reinforcement can markedly enhance environmental tolerance. Conversely, coatings relying solely on a rudimentary wax-based roughness layer typically exhibit constrained heat resistance, abrasion resistance, and operational longevity [40,201]. Such evidence underscores that the determinant of genuine sustainability is not the nominal descriptor “natural wax,” but the aggregate fabrication and maintenance expenditure required to secure the target service life. In essence, the ecological merit of a material is indexed to the efficiency with which it achieves durable functionality, rather than the provenance of its feedstock.

Concomitantly, the pursuit of enhanced functional stability invariably necessitates increased formulation complexity and processing sophistication. The food-grade anti-adhesive coating reported by Ding et al. [181] exemplifies this trend: rather than a monolithic natural wax layer, it comprised a ternary system of food-grade paraffin wax, PDMS-modified attapulgite, and a food-grade silicone binder, explicitly engineered to bolster mechanical integrity and chemical resistance. This case illustrates a critical transition—as materials progress from laboratory-scale demonstrations of liquid repellency to reliable operation under industrial food-processing regimes, sophisticated modification and interfacial bonding strategies become indispensable. While such complex formulations can extend operational longevity, they concurrently elevate the embedded costs associated with fabrication, quality control, and comprehensive safety assessment (including migration and toxicological profiling) [206,207]. This juxtaposition reveals a prevalent “green paradox” in food-contact super-liquid-repellent materials: regulatory and marketing labels disproportionately emphasize intrinsic feedstock attributes (e.g., “natural”,“fluorine-free”), whereas true sustainability is dictated by extrinsic factors—namely, process intensity, functional durability, and maintenance frequency. Consequently, sustainability evaluations must transcend binary compositional queries (e.g., “Is it natural?”). The pivotal question should instead be: Does the cumulative resource consumption (energy, materials, and capital) over the target service life represent a genuine net reduction compared to conventional alternatives?

### 3.3. Migration Risk: From “Whether Migration Occurs” to “What Migrates”

For food-contact materials, risk assessment hinges not solely on compliance with overall migration limits (OML), but critically on the elucidation of migrant chemical identity, origin, and toxicological relevance. Both intentionally added substances (IAS) and NIAS can be released during fabrication, operational use, and material aging. NIAS—encompassing synthesis by-products, impurities, and degradation products—pose a particular analytical challenge: they are often numerous, structurally uncharacterized, and lack commercial analytical standards, rendering them a significant blind spot in current regulatory frameworks and risk research [208,209]. As emphasized in the consensus statement by Muncke et al. [42], food-contact materials constitute a major exposure route for both established hazards and the vast array of chemicals for which toxicological data remain scarce or nonexistent; consequently, existing safety paradigms fail to comprehensively capture this complex exposure landscape. Therefore, inferring material safety exclusively from the food-grade status of raw feedstocks or edible substrates—without empirical migration data and exposure validation—leaves a critical evidentiary gap in the risk assessment dossier, rendering any claim of safety scientifically incomplete.

PFAS-related research unequivocally demonstrates that safety assessments must extend beyond compositional labels to incorporate empirical migration evidence. In a pivotal study investigating PFAS-containing paper food-contact materials under thermal stress, Lerch et al. [44] reported that migration levels of 6:2 fluorotelomer alcohol (6:2 FTOH) peaked at 11.3 ng/g food. Subsequent dietary exposure modeling revealed that estimated intake in children ranged from 1.06 to 5.67 ng/kg body weight (bw)/day—significantly exceeding the EFSA-derived safety threshold of 0.63 ng/kg bw/day applied in that analysis. This finding mandates a paradigm where oil repellency and anti-adhesion functionalities are never evaluated in isolation from realistic migration scenarios. When variables such as temperature, food matrix composition, and authentic contact duration are integrated, the purported functional advantages of a material must be rigorously weighed against quantifiable exposure risks. Reinforcing this caution, Tsochatzis [43] highlighted that the identification and quantification of NIAS remain severely constrained by the paucity of analytical standards, incomplete spectral databases, and the inherent structural diversity of degradation products. Consequently, the absence of detectable regulatory exceedances—or even a “non-detect” result—cannot be equated with the absence of risk; rather, it often reflects the current analytical inability to characterize the full spectrum of migrating species.

Critically, transitioning to natural or edible material systems does not obviate migration concerns [210,211,212]; rather, it transposes the chemical identities of migrants and exacerbates analytical complexities. Edible and active coating formulations, for instance, routinely incorporate plasticizers, emulsifiers, antimicrobial agents, and nanocomposite fillers to optimize film-forming ability and functionality [41,213,214,215]. Consequently, rigorous verification must extend beyond inertness to probe the stability of these additives under aggressive food matrices (e.g., acidic, high-ethanolic, or lipid-rich environments) and ascertain whether they mobilize into foods as low-molecular-weight leachates, ionic species, or particulate matter. Migration assessment [33,44,167,168,216], therefore, must pivot from validating superficial “green” nomenclature to interrogating the fundamental triad: which chemicals migrate under authentic conditions of use, to what extent, and whether they engender novel exposure pathways. To achieve this, the evaluation of food-contact super-liquid-repellent surfaces must evolve from simplistic compositional labeling to an integrated, systems-level judgment encompassing material synthesis, fabrication processes, operational service life, and empirical migration profiles. Without such a holistic framework, the “green” moniker remains tethered to mere nomenclature—a semantic veneer rather than substantive evidence of genuine risk mitigation.

Migration behavior in nanofilled food-contact systems is characterized not merely by the extent of mass transfer, but by the complex interplay of migration speciation—encompassing particulate matter, dissolved ions, and degradation products—as well as divergent interfacial evolution pathways. To systematically compare these characteristics under relevant food-simulating conditions, Table 1 synthesizes recent data on representative nanofiller systems, highlighting their functional performances. As summarized in Table 1, nanofiller systems exhibit starkly divergent in functional performance due to migration modalities. Certain fillers, such as nanodiamonds [141,217], primarily undergo particulate migration, maintaining their structural integrity upon release. In contrast, frameworks like ZIF-8 demonstrate disintegration-type migration [218,219], wherein the host structure collapses, resulting in the concurrent release of metal cations and organic ligands. This dichotomy underscores that migration is not a monolithic process; rather, it is a multipath evolution governed jointly by the intrinsic structural stability of the nanomaterial and the physico-chemical stresses imposed by the interfacial environment. Conventional bulk migration assays, which report only total extractable mass, are inherently incapable of discerning these distinct mechanistic pathways. Consequently, migration assessment must transcend mere quantitative detection to achieve mechanistic identification. This progression reinforces a central thesis of this review: evaluating food-contact materials solely on the basis of migration levels is insufficient. A comprehensive risk assessment mandate demands the integrated consideration of migrant speciation and the underlying interfacial processes that dictate their release.

## 4. Dynamic Evaluation Framework for Real Food Environments

Section 2 and Section 3 elucidated the fundamental mismatch between conventional static evaluation frameworks and the operational realities of food processing, alongside the latent environmental and health burdens obscured by prevailing “green” labeling practices. To confront these critical gaps, Section 4 advances a dynamic, multidimensional evaluation framework tailored to real-world food environments. This framework is structured around four pivotal pillars: (i) a paradigm shift in evaluation logic from endpoint-centric to process-resolved assessment; (ii) the establishment of mechanistically relevant metrics that capture interfacial evolution; (iii) a tiered, risk-informed screening workflow to prioritize candidate materials; and (iv) a critical discussion on the scalability and techno-economic feasibility of industrial implementation.

### 4.1. Shift in Evaluation Logic: From Static to Dynamic

Owing to the inherent mismatch between conventional static evaluation protocols and the dynamic operational realities of food processing, the central paradigm shift lies not in expanding the repertoire of static assays, but in transitioning from endpoint-focused assessments to process-oriented characterization. Conceptually, this entails redirecting the evaluative focus from the instantaneous wetting state to the interfacial evolution trajectory and its underlying failure mechanisms. Within this framework, evaluation metrics should be explicitly mapped to distinct failure pathways. Specifically, protein fouling can be quantified via contact angle hysteresis or retention to probe interfacial stability; lipid/oil fouling is best described by the penetration depth and kinetics within surface microtextures; microbial fouling can be characterized by the critical biofilm formation time, denoting the threshold of interfacial colonization; meanwhile, the Cassie-to-Wenzel transition time serves as a direct metric of interfacial energy stability [1,58,61,69]. Therefore, the benchmark for super-liquid-repellent materials should extend beyond merely achieving a high contact angle to addressing when and how failure occurs. This shift transforms evaluation from a singular performance descriptor into a multidimensional framework spanning time, structure, and chemistry. Ultimately, this provides a clear mechanistic basis for establishing dynamic metrics and steering targeted material innovation.

Building upon the foregoing analysis of temporal, chemical, and mechanical failure mechanisms, it is imperative to translate these disparate process-level insights into operational evaluation metrics. Conventional criteria predicated on static contact angles and discrete abrasion resistance fail to capture the critical failure pathways prevalent in authentic food-processing environments. To address this gap, the present review proposes a dynamic evaluation framework tailored to realistic food-processing scenarios, structured around the concept of interfacial evolution (Table 2). This framework facilitates a paradigm shift from mere performance reporting to mechanistic correlation.

As delineated in Table 2, the cornerstone of this dynamic framework is the explicit, one-to-one mapping between quantitative metrics and specific failure mechanisms. Protein fouling is probed via contact angle hysteresis/retention, reflecting interfacial destabilization induced by protein conformational rearrangements. Lipid fouling is characterized by the penetration kinetics into surface microtextures. Microbial contamination is quantified by the critical timescale for biofilm maturation, marking the threshold of complete interfacial colonization. In contrast to conventional static indicators, this integrated system not only enables precise performance quantification but also elucidates the dominant mechanisms governing performance degradation. Consequently, it offers actionable guidance for targeted material optimization. Furthermore, the incorporation of resistance to multi-component food matrices and migration indices extends the scope of evaluation beyond functional efficacy to encompass application safety and long-term sustainability. Collectively, this metric system establishes a process-based evidentiary chain that bridges endpoint assessment and mechanistic understanding, serving as a vital conduit between fundamental interface science and industrial engineering applications.

### 4.2. Tiered Screening Workflow: An Operable Path from Research to Industry

To ensure the practical utility of the proposed dynamic evaluation metric system, merely defining metrics is inadequate; it must be integrated with a tiered decision-making framework aligned with the material development pipeline. This hierarchical screening workflow fundamentally redefines material assessment—shifting the paradigm from binary pass/fail judgments based on isolated tests to a cumulative, evidence-based decision-making process. Such stratification is essential because food-induced interfacial fouling is inherently a multistage evolutionary process (e.g., protein adsorption → conformational rearrangement → irreversible fouling layer formation), wherein distinct mechanisms dominate at each stage. Consequently, a phased screening approach is indispensable for progressively de-risking candidate materials and accurately discerning their true application potential [227].

Tier 1: Basic Screening—Rapid Identification of “Obviously Failing Materials”

In the nascent stages of material development, the primary objective is not yet an in-depth mechanistic inquiry, but rather to ascertain whether a candidate possesses the basic viability to advance to subsequent phases. Consequently, Tier 1 screening should employ low-cost, high-throughput methodologies to rapidly disqualify materials exhibiting premature failure under food-mimetic interferential conditions.

This approach is predicated on the understanding that proteins and other surface-active components in food matrices can rapidly occupy interfacial sites, thereby triggering wetting state transitions. Such interactions typically manifest as a significant reduction in the static contact angle and a concomitant increase in adhesion [1,69]. Empirical evidence indicates that, in protein-laden environments, certain super-liquid-repellent surfaces suffer a contact angle decay exceeding 20° within 24 h; this geometric deterioration is often coupled with a pronounced elevation in the roll-off angle, signifying a deleterious transition from a low-adhesion (self-cleaning) regime to a high-adhesion (fouling-prone) state [1].

Therefore, the mandate of Tier 1 is to efficiently down-select candidates through simplified, yet diagnostic, metrics. For instance, post-fouling contact angle retention serves as a robust proxy for interfacial stability under realistic perturbations, obviating the need for sophisticated instrumentation while enabling effective elimination of non-viable formulations.

Tier 2: Mechanistic Analysis—From “Performance Decline” to “Failure Pathway Identification”

Upon passing the initial screening, it becomes insufficient to merely document performance degradation; the dominant failure mechanism must be elucidated to rationally guide material refinement. Consequently, the core mandate of Tier 2 is to establish a definitive correlation between quantitative performance metrics and specific failure pathways.

Typical food-induced interfacial fouling encompasses a spectrum of physicochemical processes: irreversible adsorption driven by protein conformational rearrangement; structural occlusion resulting from lipid penetration into micro/nanoscale textures; and interfacial restructuring triggered by multicomponent competitive adsorption [1,58,61,69,80,81,82,83,84,85,228]. These pathways underscore that material failure transcends simple wetting deterioration, manifesting instead as a structured evolution governed by a predominant mechanism. Therefore, Tier 2 necessitates the deployment of metrics capable of resolving these underlying processes—such as lipid penetration kinetics/depth and protein-induced interfacial destabilization—rather than relying solely on macroscopic contact angle variations. By mapping performance loss onto discrete mechanistic origins, this stage transforms an otherwise ambiguous failure into a diagnosable and controllable engineering challenge, thereby providing unambiguous directives for targeted material design.

Tier 3: Hidden-Cost Assessment—From “Functionally Feasible” to “Practically Applicable”

While superior functional performance is a prerequisite, it does not guarantee practical utility. Therefore, the core mandate of Tier 3 screening is to evaluate the systemic liabilities related to safety and sustainability, thereby bridging the gap between laboratory efficacy and engineering feasibility.

Regarding safety, the critical inquiry shifts from the mere occurrence of migration to the chemical speciation of migrants and their associated toxicological risks. Current evidence indicates that nanofillers can infiltrate food matrices either as intact particulates or as dissolution products, with hazards contingent upon particle size, dosage, and physicochemical composition [220,225,229,230,231,232,233,234,235,236]. For instance, nano-sized entities exhibit enhanced propensity to traverse biological barriers, whereas structurally compromised coatings may undergo concurrent release of multiple leachates. Concurrently, in the environmental dimension, the ecological credentials of a material must be assessed via a life-cycle perspective rather than isolated attributes of raw materials. Notably, short-lived coatings often necessitate frequent refurbishment; consequently, their cumulative energy expenditure and waste streams may surpass those of more durable alternatives—a phenomenon termed the “green paradox” [40].

Consequently, Tier 3 prioritizes holistic metrics, such as migration profiles and life-cycle impact assessments. These indicators serve to distinguish materials possessing genuine long-term application value from those merely satisfying transient performance benchmarks.

The aforementioned three-tiered strategy culminates in a hierarchical evaluation pathway progressing from phenomenology to mechanism, and ultimately to system-level validation:

Tier 1: Rapid exclusion of candidates exhibiting premature failure (Phenomenological Level).

Tier 2: Elucidation of the dominant failure mechanisms (Mechanistic Level).

Tier 3: Validation of genuine application potential regarding safety and sustainability (Systems Level).

The pivotal advancement offered by this workflow is its transformation of material assessment from reductionist single-metric judgments into a multilayered, evidence-based decision-making framework. By bridging the gap between isolated laboratory phenomena and complex engineering realities, this approach provides a robust roadmap for the effective translation of super-liquid-repellent materials from benchtop discovery to industrial deployment.

### 4.3. Challenges for Industrial Scale-Up and Material Selection Strategies

The industrial translation of super-liquid-repellent materials in the food sector hinges not merely on achieving static benchmarks—such as a contact angle > 150° or a roll-off angle < 10° in controlled laboratory settings—but on their capacity to concurrently satisfy multifaceted constraints in real-world food-contact environments. These constraints encompass food safety compliance, structural integrity, interfacial durability, and process compatibility [237]. Central to industrial applicability is the concept of performance retention throughout repeated fouling–cleaning–refouling cycles. Typical food-processing regimes, involving prolonged alkaline washing (pH 10–12) and thermal rinsing (60–90 °C) [238], can synergistically degrade low-surface-energy layers, inflict microstructural damage, and exacerbate fouling accumulation. Empirical studies corroborate that coatings exhibiting an initial contact angle > 155° can degrade rapidly to <120° following cyclic alkaline exposure [205]. Such findings underscore that static metrics are inadequate proxies for long-term engineering stability; consequently, the engineering viability of these materials must be predicated on the evaluation of their dynamic service behavior.

#### 4.3.1. Material System Selection and Performance Trade-Offs

Divergence in performance among material classes stems fundamentally from distinct strategies for interfacial energy regulation and structural stabilization. Synthetic polymers, exemplified by polydimethylsiloxane (PDMS), leverage intrinsically low surface energy coupled with flexible chain segments. This viscoelastic nature allows them to buffer external mechanical stress through deformation, thereby preserving interfacial integrity [239]. Experimental evidence demonstrates that such systems maintain high contact angles under high-frequency mechanical agitation [240], rendering them suitable for high-flush or repetitive cleaning scenarios. Conversely, inorganic nanomaterials (e.g., SiO_2_, ZnO) primarily serve to construct hierarchical roughness, with functionality reliant on the stability of the entrapped air cushion (Cassie state) [241]. However, their engineering robustness is critically dependent on interfacial anchoring strength. Reliance on weak physical adsorption renders these structures susceptible to detachment under fluid shear, precipitating catastrophic failure [242]. Thus, in an engineering context, surface efficacy should be defined by the maintainability of the micro-nano architecture over time, rather than its pristine morphological characteristics.

Bio-based materials offer distinct advantages in terms of renewable sourcing and inherent food-contact compatibility. Nevertheless, their operational envelope is often circumscribed by limited thermodynamic stability. For instance, wax-based matrices are prone to structural reorganization under moderate thermal loads, leading to irreversible deterioration of interfacial function [243,244,245]. Even ostensibly edible systems [245] built from FDA-approved wax/gum/gelatin still require solvent-assisted deposition and thermal annealing, and their safety dossier terminates at static repellency-illustrating that ‘edible’ substrates do not exempt the composite from migration and dynamic-fouling scrutiny. Consequently, these materials are best suited for low-temperature or short-duration applications, where long-term stability under thermal or cleaning stress is not a prerequisite.

Biomimetic coatings derived from renewable or biologically derived components represent an emerging frontier that transcends the “natural = sustainable” narrative. For example, cholesterol-containing polymer brushes have been used to prepare temperature-responsive interfaces that undergo reversible conformational transitions [246]. While developed for non-food applications, this concept illustrates how biologically derived components can confer not only renewable origin but also specific responsive surface functions. Other notable examples include mussel-inspired polydopamine coatings (catechol-mediated adhesion) [247,248], saccharide-based antifouling layers [249], and stimuli-responsive biohybrids [250,251]. These systems align with the paradigm shift advocated in this review—from passive repellency to active modulation of fouling evolution.

Given the intrinsic limitations of monolithic systems, composite materials represent an increasingly pragmatic avenue for industrial deployment. Their core design principle involves the synergistic unification of structural robustness and interfacial functionality via hybrid scaffolds and low-surface-energy components. A pertinent example is the wax/attapulgite system, where an inorganic skeleton provides mechanical reinforcement while mitigating lipid penetration [181,252]. The engineering merit of composites lies not in mere performance summation, but in their capacity to modulate fouling pathways. However, this sophistication introduces challenges related to fabrication complexity and batch-to-batch consistency.

Beyond static contact angle, a comprehensive dynamic evaluation should incorporate parameters that capture the thermodynamic and kinetic aspects of competitive interfacial adsorption: (i) surface free energy (SFE) decomposed into polar and dispersive components via the Owens–Wendt or van Oss–Chaudhury–Good models, which predicts the thermodynamic driving force for protein and lipid adsorption [253,254]; (ii) advancing/receding contact angles and hysteresis (Δ*θ* = *θ*_a_ − *θ*_r_), reflecting chemical heterogeneity and topological complexity at the three-phase contact line [255,256]; (iii) work of adhesion (*W*_a_), quantifying the interfacial binding strength between foulants and the surface [257,258]; and (iv) dynamic contact angle measurements under controlled flow or impact conditions [259]. These parameters are particularly critical when evaluating protein and lipid fouling, as they resolve the mechanistic drivers of failure that a single static angle cannot capture.

#### 4.3.2. Persistent Challenges and Scenario-Based Selection

From an engineering vantage point, three principal barriers impede widespread adoption: (i) insufficient long-term stability (inability to sustain the Cassie state under chemical cleaning, thermal cycling, or mechanical shear) [1,61,238]; (ii) limited compatibility with complex food matrices (poor repellency against proteinaceous, high-fat, or viscous media despite efficacy against water/simple oils) [1,112]; and (iii) incomplete safety dossiers concerning component migration, particulate shedding, and non-intentionally added substances (NIAS) [43,208,220,229,230,231,232,233,234,235,236]. Collectively, these deficiencies explain why many super-liquid-repellent materials remain confined to the proof-of-concept stage.

Consequently, material selection must evolve from a categorical preference to a scenario-based, multi-parametric optimization framework. For instance, inorganic-polymer hybrids may be prioritized for high-shear, high-cleaning-frequency environments, whereas natural waxes or edible coatings may suffice for short-term, low-temperature packaging. Systems incorporating antimicrobial actives necessitate concurrent evaluation of migration safety and release kinetics. In essence, the pivotal question is not which material is universally “best,” but which achieves the optimal Pareto frontier balancing repellency, durability, safety, processability, and sustainability within a specific operational context.

As delineated in Table 3, distinct material systems exhibit clear performance trade-offs. Synthetic polymers offer superior mechanical resilience; inorganic nanomaterials excel in roughness fabrication; bio-based/edible coatings are attractive for transient applications; and composites potentially offer the most balanced engineering profile. However, no singular material class satisfies all requisites. Selection must therefore be application-driven, integrating multiple metrics rather than pursuing isolated performance peaks.

#### 4.3.3. Emerging Material Classes: From Passive Repellency to Active Modulation

The foregoing analysis underscores that conventional material strategies—synthetic polymers, inorganic nanomaterials, and simple bio-based coatings—each suffer from distinct, often irreducible limitations when evaluated against the dynamic, multidimensional criteria of real food environments. To transcend the passive-repellency paradigm, recent research has pivoted toward material classes capable of actively modulating interfacial fouling evolution. The following analysis summarizes six representative emerging systems, assessed against the key failure pathways identified in this review: protein adsorption, lipid infiltration, microbial colonization, mechanical degradation, and migration risk.

Polymer brushes graft dense, flexible chains (e.g., PEG, PNIPAM, polyacrylamides) onto a substrate, creating a dynamically fluctuating, hydrated steric barrier that suppresses protein approach and conformational unfolding [260,261,262,263,264]. Their fouling resistance is exceptional—contact-angle hysteresis remains low even after prolonged protein exposure—but their mechanical resilience under fluid shear and abrasive cleaning is limited by chain detachment or cleavage. Furthermore, the synthesis of densely grafted brushes at industrial scale remains challenging, and residual unreacted monomers pose potential migration concerns.

Zwitterionic coatings (e.g., poly(carboxybetaine methacrylate), poly(sulfobetaine methacrylate)) achieve ultra-low fouling through a fundamentally different mechanism: their paired cationic–anionic moieties bind water molecules with exceptional tenacity, forming a hydration layer that proteins cannot displace without a severe desolvation penalty [265,266,267,268]. This confers resistance to nonspecific adsorption that rivals or exceeds PEG-based systems, even against complex, multicomponent food matrices. However, the scalability of zwitterionic coatings is constrained by synthesis cost, and their long-term stability under alkaline cleaning (pH 10–12) requires further validation. Migration of low-molecular-weight oligomers is also a safety consideration that must be addressed for food-contact approval.

Stimuli-responsive surfaces undergo reversible changes in wettability, topography, or surface charge upon exposure to external triggers (temperature, pH, light, or enzyme presence) [269,270,271]. For instance, PNIPAM-based coatings exhibit a lower critical solution temperature (LCST) transition [272,273] that can be exploited to release adherent foulants upon cooling. The conceptual alignment with the dynamic evaluation framework is clear: such surfaces do not merely resist fouling—they actively reset interfacial conditions. Nevertheless, response times (often minutes to hours) are typically slower than the timescale of fouling initiation (seconds), and the incorporation of responsive elements into mechanically robust, food-safe matrices remains an unsolved engineering problem.

Self-healing systems [274,275,276,277,278] (e.g., Diels–Alder networks, disulfide-exchangeable polymers, microcapsule-embedded matrices) recover interfacial functionality after mechanical damage, thereby extending service life and reducing the frequency of recoating—a direct mitigation of the “green paradox” discussed in Section 3.2. However, the healing agents themselves (e.g., encapsulated low-surface-energy fluids, unreacted monomers) may migrate into food matrices, and the long-term toxicological profile of self-healing components is largely unevaluated. Thus, while self-healing addresses the mechanical dimension of the framework, it introduces new questions in the safety dimension.

LIPS/SLIPS [279,280,281,282,283] impregnate a chemically matched lubricating liquid into a porous or textured scaffold, creating a mobile, self-smoothing interface that sheds proteins, lipids, and microbes with minimal adhesion. LIPS perform exceptionally well against high-viscosity and particulate-laden food fluids, and the lubricating layer can spontaneously refill minor defects, conferring a degree of self-healing. The critical limitation for food applications is lubricant depletion and leaching: under thermal cycling, surfactant exposure, or high-pressure cleaning, the infused liquid can be displaced, triggering a catastrophic loss of function and introducing a new migration pathway. The safety assessment of LIPS must therefore encompass not only the solid scaffold but also the identity, toxicity, and migration kinetics of the lubricating phase.

Organic–inorganic hybrid coatings [181,284,285,286] (e.g., wax/attapulgite, PDA@SiO_2_, MOF/cellulose) combine structural reinforcement from inorganic scaffolds with interfacial functionality from organic modifiers, offering a balanced engineering profile across multiple evaluation dimensions. The wax/attapulgite system, for example, leverages an inorganic skeleton to resist lipid penetration while maintaining a low-surface-energy outer layer [181]. However, fabrication complexity, batch-to-batch consistency, and the migration of nanofillers or degradation products remain persistent challenges.

Collectively, these emerging classes illustrate a central thesis of this review: the future of food-contact super-liquid-repellent materials lies not in maximizing a single static metric, but in co-designing surfaces that interrupt specific failure pathways. No single material satisfies all criteria simultaneously. Instead, material selection should follow a scenario-driven optimization: for high-shear, high-cleaning-frequency lines, polymer–inorganic hybrids with mechanical reinforcement may be prioritized; for short-duration, low-temperature packaging, bio-based or zwitterionic coatings may suffice; for applications where fouling recovery is paramount, stimuli-responsive or self-healing systems warrant exploration—provided their migration safety is validated. This nuanced, mechanism-aware approach to material design and selection is the ultimate objective of the dynamic evaluation framework proposed herein.

#### 4.3.4. Rethinking the “Green” Material Dichotomy

Discussions surrounding sustainable materials often devolve into a binary comparison between bio-based/edible coatings and fluorinated counterparts. However, this oversimplification obscures critical nuances in performance and risk profiles. To circumvent overgeneralization, Table 4 provides a comparative analysis based on repellency, durability, safety, and sustainability metrics.

The data reveal that bio-based and fluorinated materials do not share a simple substitution relationship; rather, they embody multidimensional trade-offs. Fluorinated variants retain distinct advantages in low-surface-energy liquid repellency, mechanical tenacity, and thermal stability. Bio-based alternatives, while advantageous in feedstock renewability, often suffer from abbreviated service lives necessitating frequent renewal. This recurrent replacement can paradoxically amplify cumulative environmental burdens—a manifestation of the “green paradox” previously discussed. Therefore, material evaluation must transcend simplistic labeling (e.g., “bio-based” [287,288] or “fluorine-free”) and instead focus on integrated lifecycle performance under equivalent service-life conditions.

**Table 4:** Multidimensional comparison between bio-based/edible materials and fluorinated materials for food-contact super-liquid-repellent applications.

Dimension	Bio-Based/Edible Materials	Fluorinated Materials
Liquid repellency	Repellency to water and high-surface-tension food liquids, reducing food residues [218,289]	Can repel water and various low-surface-tension oils [290]
Mechanical stability	Moderate; requires inorganic frameworks, binders, or composite structures for reinforcement [218,289]	Generally high, with strong chemical inertness and low adhesion [290]
Thermal stability	Low to moderate, limited by wax softening, melting point, and matrix stability [218,289]	Relatively high; can maintain non-stick behavior at elevated temperatures [290]
Food-contact safety	Potentially high, but need migration and toxicological evaluation [218,289]	Controversial; PFAS may migrate from food-contact materials into foods [291,292]
Migration behavior	May release small molecules, active compounds, degradation products, or nanofillers [223,224,226]	May release PFAS or PFAS precursors [291,292]
Fabrication cost	Low; raw materials readily available, and processes relatively simple [218,219]	High; required specialized fluorinated components and surface treatments [290,291]
Service life	Short; susceptible to heat, abrasion, oil/lipid penetration, and cleaning cycles [218,219,289]	Long; generally stronger chemical and thermal resistance [290,291]
Life-cycle environmental impact	Uncertain; short service life and frequent replacement may weaken their green advantages [190]	Requires strict evaluation; PFAS exhibit environmental persistence [291,292]

## 5. Summary and Outlook

This review establishes that conventional static metrics—such as contact angle and dry abrasion resistance—are fundamentally inadequate for predicting the performance of super-liquid-repellent materials in real food-processing environments [293,294]. We demonstrate that interfacial failure is governed by sub-surface, time-dependent evolution, including protein conformational rearrangement, lipid infiltration into micro/nanostructures, and progressive biofilm formation, which precede macroscopic wetting changes. Consequently, evaluation paradigms must shift from verifying initial repellency to quantifying long-term interfacial stability, cleanability, and safety. Furthermore, simplistic labeling (e.g., “fluorine-free” or “bio-based”) must be superseded by an integrated, lifecycle-aware framework—encompassing material composition, fabrication, service aging, migration, and end-of-life fate—to accurately reflect true application risks.

Despite proposing a dynamic, process-oriented evaluation framework, significant challenges remain before industrial translation. Current dynamic metrics lack standardized definitions and are highly sensitive to boundary conditions, while most mechanistic insights derive from oversimplified mono-component models that fail to capture the multivariate complexity of real food matrices. Additionally, advanced in situ characterization techniques face barriers in reproducibility and throughput, and the quantitative linkage between laboratory-scale metrics and actual engineering service life remains poorly defined. Addressing these gaps requires moving beyond descriptive parameters toward unified protocols and closed-loop validation platforms.

Future research must pivot from pursuing passive, extreme liquid repellency toward the active modulation of fouling evolution through responsive surfaces and self-healing interfaces. This necessitates a concurrent focus on five key areas: (i) establishing scenario-based standardized testing protocols; (ii) designing materials that interrupt specific failure pathways; (iii) integrating machine learning with in situ spectroscopy for rapid industrial screening; (iv) constructing lifecycle databases to enable multi-criteria decision-making beyond single-performance metrics; and (v) validating material performance under realistic, long-term processing conditions. Ultimately, bridging the gap between academic innovation and industrial deployment requires a holistic paradigm where material design, dynamic evaluation, and safety assessment are co-developed to meet the rigorous demands of complex food environments.

## Figures and Tables

**Table 1:** Functional performance of different nanofiller-food-contact material systems in food simulants.

Nanofiller Type	Material System	Food Simulants	Migration Conditions	Key Findings
Nanodiamonds (NDs)	PDA–ND/PVC coating; PVC/PDMS–ND membrane	Water, 3% acetic acid, 10% ethanol, 95% ethanol	Set according to the target food-contact scenario	WCA reaching 151.9–159.5°, while reducing the adhesion of foodborne pathogens [141,217]
Silica (SiO_2_)	Biowax–SiO_2_ coating; PLA/PCL/PHBV–SiO_2_ paper-based coating	Water, acidic simulants, ethanol-based simulants	Ambient or accelerated migration conditions	SiO_2_-matrices texture improve liquid repellency of paper-based materials, and reduce food residues [220,221,222]
Metal–organic frameworks (MOFs, ZIF-8)	Carvacrol-loaded ZIF-8/cellulose paper; PDA@ZIF-8/cellulose paper	Ethanol-based simulants, high-humidity environments	Room-temperature storage or accelerated migration conditions	ZIF-8 framework dissociation, Zn^2+^ release, and loaded active compounds migrated [218,219]
Halloysite nanotubes (HNTs)	Oil/carvacrol-loaded HNTs–LDPE/EVA film; HNT–biopolymer film	Water, ethanol-based simulants, real foods	Set according to packaging storage or simulated migration tests	HNTs carry active compounds and enable sustained release; polymer embedding reduces nanofillers migration [223,224]
Silver nanoparticles (AgNPs)	Chitosan–AgNP film; polymer–AgNP material	3% acetic acid, water, 10% ethanol, 50% ethanol	25–70 °C or long-term storage conditions	AgNPs: Ag^+^ release in acidic simulants; migration assessment should distinguish particulate silver, ionic silver, and total silver content [225,226]

**Table 2:** Dynamic evaluation metrics for super-liquid-repellent materials.

Evaluation Dimension	Traditional Static Metric	Dynamic Evaluation Metrics	Proposed Acceptance Criteria
Baseline liquid repellency	Static contact angle (CA) > 150°	Initial CA with water, vegetable oil, and non-Newtonian fluids [22,46,47,48]	>150° (water), >140° (oil)
Time-dependent antifouling performance	None	CA retention after 24 h of protein fouling [73]	CA decay < 15°
	None	Half-times of penetration of oil/lipid	>12 h
	None	Critical time for biofilm formation [86]	>72 h = excellent; 24–72 h = moderate; <24 h = failure
Resistance to multicomponent media	Pure-water test	Surfactant-induced C-W transition time [69]	>48 h
	Single liquid	Repellency for multicomponent simulants	CA > 140° for multicomponent simulants
Mechanical robustness	Sandpaper abrasion	Performance after fluid-shear/chemical-attack	CA > 140° after cycling
	Dry abrasion	Resistance to high-temperature cleaning	CA > 140° after 80 °C/30 min

**Table 3:** Comparison of common super-liquid-repellent coating materials in terms of performance, mechanism, and application features.

Material Type	Representative Materials	Main Advantages	Key Limitations
Synthetic polymers	Polyurethane, epoxy resin, PDMS	High mechanical stability; good impact resistance and fatigue resistance; adaptable to complex conditions	Non-degradable; food-contact safety of some systems requires validation
Inorganic nanomaterials	SiO_2_, ZnO, TiO_2_, attapulgite	Large specific surface area; tunable micro/nano structures; potential antibacterial function	Easy aggregation; dependence on interfacial anchoring; high detachment risk
Bio-based materials	Beeswax, carnauba wax, chitosan, starch	Renewable sources; potential food-contact friendliness; simple fabrication	Low thermal stability; weak mechanical strength; easy structural collapse
Composite materials	Wax + attapulgite, PDA + SiO_2_, MOF + cellulose	Strong tunability; balance between stability and functionality; adaptability to complex food systems	Complex fabrication; higher cost; difficult consistency control

## Data Availability

No new data were created or analyzed in this study. Data sharing is not applicable to this article.
